# To the Editors of the Medical and Physical Journal, No. 1, Paternoster-Row, London

**Published:** 1815-05

**Authors:** Thomas Vaughan

**Affiliations:** Cardiff


### Medical and Physical Journal.

5 OF VOL. XXXIII.] MAY, 1815. [NO. 195.

"For many fortunate discoveries in medicine, and for the detection of numerous errors, the world is indebted to the rapid circulation of Monthly Journals; and there never existed any work to which the faculty in EUROPE and AMERICA were under deeper obligations than to the Medical and Physical Journal of London, now forming a long, but an invaluable series."—RUSH.

*To the Editors of the Medical and Physical Journal,*
*No. 1, Paternoster-row, London.*

GENTLEMEN,

I HAVE been in the habit of taking your Journal for several years, but my bookseller has now sent me a different publication, saying yours is discontinued: I beg to know if that is the case, as I certainly give your's a decided preference to any I have seen.

I am, Gentlemen,

*Cardiff,* Your obedient servant,

*March* 23, 1815. THOMAS VAUGHAN.

THE Proprietors feel grateful for the above communication; nor can they wonder at the delicacy of Dr. Vaughan in declining to answer their inquiries after the name of the bookseller. They find no difficulty in guessing his motives. It affords them another opportunity of expressing their gratitude for increased communications; and to assure the public, that nothing can be further from their intentions than to relinquish a concern, the encouragement of which has been found progressively greater every succeeding year.

*N.B.—The demand for complete sets, and for single numbers, has been so considerable since the opening on the continent, that very few are left of many of the early numbers: we would, therefore, recommend our friends who have imperfect sets, to complete them without delay, to prevent disappointment.*

